# Predicting the functional independence during the recovery phase for poststroke patients

**DOI:** 10.1002/nop2.335

**Published:** 2019-07-11

**Authors:** Sadeq AL‐Fayyadh

**Affiliations:** ^1^ Adult Nursing Department, School of Nursing University of Baghdad Baghdad Iraq

**Keywords:** functional capacity, independence, nursing, rehabilitation, stroke, stroke‐related‐self‐efficacy

## Abstract

**Background:**

Successful recovery of stroke survivors can be challenging. However, when targeted functional capacities are predicted early in the recovery phase, necessary nursing intervention can be initiated aiming at supporting the client moving forward in the rehabilitation journey.

**Aim(s):**

This study aimed to evaluate stroke self‐efficacy of poststroke patients and identify the differences in stroke self‐efficacy level among some relevant variables.

**Design:**

A descriptive cross‐sectional design was employed to achieve the aforementioned objectives.

**Methods:**

A purposive sample of 207 poststroke patients who were recovering from stroke in three major teaching hospitals at Baghdad city were recruited to participate in the study. Data collection process started 3 November 2016 –15 May 2017. Inclusion criteria embraced stroke patients who were adult, have Glasgow Coma Scale score 14–15, capable of giving written or verbal consent. The modified version of the stroke self‐efficacy questionnaire was used for data collection.

**Results:**

A significant statistical difference at the *p*‐value ≤ 0.05 level, in stroke self‐efficacy, was verified among subjects’ age, residency, stroke incidence and patient's knowledge about his/her stroke medical diagnosis.

## 1 INTRODUCTION

Both in developing and developed nations, non‐communicable chronic diseases (NCDs) are a substantial public health threat of the 21st century (Beech, 2013). Forty million people every year are losing their lives as a direct result of NCDs. This constitutes 70% of the mortality rate on a global level (World Health Organization, 2017). Stroke, formerly known as cerebrovascular accident (CVA), is one of the most alarming NCDs. Stroke devastating consequences can endanger all essential facets of patient's life. Activities of daily living (ADL), social relationships and professional capacities are some examples of patient's life aspects that can be negatively influenced by both stroke's long‐ and short‐term consequences (Feigin, Norrving, & Mensah, 2017). All the previously mentioned overwhelming stroke‐related consequences can cause a substantial deterioration in the perceived client's life quality, to the extent that patients could lose confidence in their potentials and believe that they would not sustain a productive and meaningful life. Therefore, stroke management should start as soon as possible as indicated by the literature. The rehabilitation journey must commence directly after confirming the medical diagnosis, starting from managing stroke‐related life‐compromising problems (Roshanzamir, 2016). This necessitates an active engagement in fulfilling the planned rehabilitation goals of all the involved parties, particularly the patient him or herself. High stroke self‐efficacy is connected with better clinical outcome (Korpershoek, van der Bijl, & Hafsteinsdóttir, 2011). Therefore, this research was designed to assess the levels of stroke survivor's self‐efficacy in terms of its major pillars, functional capacity and self‐management. The main research questions that the study was developed to answer were as follows: What is the level of stroke self‐efficacy of poststroke patients? and What are the differences in stroke self‐efficacy level among some demographic and clinical variables of patients recovering from stroke?

## 2 BACKGROUND

The unprecedented increase of ageing population percentage can and will aggravate the situation at the level of the global public health arena. This necessitates both an effective and urgent intervention to help the huge numbers of stroke patients dealing with their multi‐dimensional health problem by facilitating a planned recovery under the umbrella of healthy transition. Therefore, extending a helping hand to stroke patients aiming at improving their functional independency is vital during the early stages of their acute illness. The literature suggested that functional independence of patients with chronic conditions such as stroke can be predicted by assessing patient's self‐efficacy (Torkia, 2014). Unfortunately, scientific inquiries that have explored self‐efficacy in a Middle Eastern stroke population are scarce, which justifies this research endeavour, aiming basically at attaining a more inclusive, clinically pertinent comprehension of self‐efficacy during the recovery phase in the aforementioned population. Self‐efficacy has been defined as person's belief in his/her ability in organizing, managing and executing the steps that are necessary to achieve the targeted goal(s) (Jones, Partridge, & Reid, 2008). Based on the aforementioned definition, it is crucial to assess stroke self‐efficacy to predict the independence level in terms of functional capacities and self‐management dimensions in poststroke patients. This would be helpful to them in terms of moving forward in the rehabilitation program. Assessing stroke patient's self‐efficacy level is the starting point of applying nursing therapeutics. As a result, it is mandatory to assess stroke patient's self‐efficacy level as an infrastructure of the rehabilitation journey. The outcome of the planned assessment would be essential in designing and implanting tailored nursing therapeutics (Tables 1, 2 and 3).

**Table 1:** Demographical characteristics and stroke‐related clinical information of patients recovering from stroke

Variable	f	%
Gender		
Male	116	56.0
Female	91	44.0
Age		
29–39	20	9.7
40–49	47	22.7
50–59	46	22.2
60–69	54	26.1
≥70	40	19.3
Residency		
Rural	73	35.3
Urban	134	64.7
Education		
Not read nor write	78	37.7
Reads & writes	23	11.1
Primary school	42	20.3
Secondary school	18	8.7
High school	30	14.5
Bachelor degree	16	7.7
Stroke type		
Haemorrhagic	44	21.3
Ischaemic	163	78.7
Stroke incidence		
1st time	168	81.2
2nd time	30	14.5
3rd time or more	9	4.3
Stroke duration		
<30 days	112	54.1
3–6 months	58	28.0
≥12 months	37	17.9
Knowledge about medical diagnosis		
Knowledgeable	55	26.6
Unknowledgeable	75	36.2
Not sure	77	37.2
Total	207	100

Note

The underlined numbers represent the highest percentages of the selected variables. In which, more than half (56.0%) of the study sample were males. More than a quarter (26.1%) of the study sample were classified as elderly individuals within 60–69 years. In terms of residency, the highest percentage (64.7%) of the study sample were suburbanites. (37.7%) of the study sample were unable to read and writes. Of equal importance, (78.7%) of the study sample were diagnosed with ischaemic stroke. More than three quarters (81.2%) of the study sample had their first stroke at the time of data collection. Time since being diagnosed with stroke was a main variable, whereas (54.1%) of the study sample categorized under the umbrella of acute stage of recovery, which was “less than 30 days”. Of equal importance, (37.2%) of the patients were not definite about their specific stroke type.

**Table 2:** Differences in Stroke Self‐Efficacy among two‐level variables

	Variable	Categories	*N*	Mean rank	Mann‐Whitney *U*	Asymp. Sig.
Self‐efficacy	Gender	Male	116	100.84	4,911.5	0.391
		Female	91	108.03		
	Residency	Rural	73	82.28	3,305.5	0.000
		Urban	134	115.83		
	Stroke type From medical records	Haemorrhagic	44	117.75	2,981.0	0.086
		Ischaemic	163	100.29		

Note

Mann–Whitney test indicates that female patients have a better stroke self‐efficacy than that of male patients. However, there is no a statistically significant difference in patients’ stroke self‐efficacy between the gender groups (*U* = 4,911.5, *p*‐value = 0.391). On the other hand, stroke patients who live in urban areas have a better stroke self‐efficacy than that of patients who live in rural areas. A statistically significant difference in patients’ stroke self‐efficacy between the residency groups (*U* = 3,305.5, *p*‐value = 0.000) was verified. Moreover, patients who were diagnosed with haemorrhagic stroke have a better stroke self‐efficacy than that of patients who were diagnosed with ischaemic stroke. However, there is no a statistically significant difference in patients’ stroke self‐efficacy between the stroke type groups (*U* = 2,981.0, *p*‐value = 0.386).

**Table 3:** Differences in Stroke Self‐Efficacy among three or more levels variables

	Variable	Categories	*N*	Mean Rank	*χ* ^2^	*df*	Asymp. Sig.
Self‐efficacy	Age	29–39	20	111.85	12.914	4	0.012
		40–49	47	129.80			
		50–59	46	95.75			
		60–69	54	94.35			
		70‐more	40	92.28			
	Educational level	Not read nor write	78	95.64	3.652	5	0.601
		Reads & writes	23	102.96			
		Elementary school	42	109.44			
		Secondary school	18	110.97			
		Preparatory school	30	104.90			
		University level degree	16	122.44			
	Knowledge about medical diagnosis	Knowledgeable	55	115.28	7.845	2	0.020
		Unknowledgeable	75	111.10			
		Not sure	77	89.03			
	Stroke incidence	1st time	168	106.89	6.945	2	0.031
		2nd time	30	103.13			
		3rd time or more	9	53.00			
	Stroke duration	<30 days	112	104.67	0.648	2	0.723
		3–6 months	58	99.36			
		>12 months	37	109.24			

Note

Kruskal–Wallis Test reveals that stroke patients who are within 40–49 years age group, with Bachelor degree, and have been affected by stroke for more than a year, have a better stroke self‐efficacy than that of other groups. Table 3 also shows that there is a statistically significant difference in patients’ stroke self‐efficacy among the age groups (*χ* ^2^ = 12.914, *df* = 4, *p*‐value = 0.012). However, there is no a statistically significant difference in patients’ stroke self‐efficacy among the educational level groups (χ^2^ = 3.652, *df* = 5, *p*‐value = 0.601), and stroke duration groups (*χ* ^2^ = 0.648, *df* = 2, *p*‐value = 0.723). Of equal importance, knowledgeable patients about their stroke type and patients who are affected by stroke for the first time in their life have a better stroke self‐efficacy than that of other groups. A statistically significant difference was detected in patients’ stroke self‐efficacy among the knowledge about stroke specific type groups (*χ* ^2^ = 7.845, *df* = 2, *p*‐value = 0.020) and stroke incidence groups (*χ* ^2^ = 6.945, *df* = 2, *p*‐value = 0.031).

## 3 METHODS

### 3.1 Study design, sample and setting

A descriptive cross‐sectional design study was conducted on 207 patients who were recovering from stroke in three major teaching hospitals at Baghdad city from 3 November 2016–15 May 2017. As it consistent with nursing studies, the study targeted an alpha level of 0.05, an effect size of 0.891, a power of 80 and a sample size of 207 (Grove, Burns, & Gray, 2013). Of equal importance, inclusion criteria embraced stroke patients who were adult have Glasgow Coma Scale (GCS) score 14–15, capable of giving written or verbal consent.

### 3.2 Data collection and tool(s)

The modified version of the stroke self‐efficacy scale (Riazi, Aspden, & Jones, 2014) was selected to achieve the study objectives. The 13‐item stroke self‐efficacy scale was found to have satisfactory feasibility and face validity to be used during the stroke recovery phase. Cronbach alpha is 0.90 signifying a satisfactory internal consistency and criterion validity is good, r = 0.803, *p* < 0.001. The stroke self‐efficacy questionnaire consisted of two sections: the first section focused on socio‐demographic characteristics and stroke‐related clinical variables. The second section included the stroke self‐efficacy scale, where 13 items focused on functional independency of stroke clients. This scale was rated based on the following numerical range (0–3). subjects in this study were given an opportunity to rate their own confidence “self‐efficacy” level based on the aforementioned scale continuum. Whereas “0” stands for “not at all confident in his or her functional independence,” “3” stands for “very confident in his or her functional independence.” The questions focused on examining patients’ confidence about their physical ability doing some tasks that maybe challenging since their most recent stroke attack. Subjects were asked to encircle the numerical value on the scale form that shows how certain they were in doing the tasks despite stroke. The level of stroke self‐efficacy was assessed and consequently classified in to three levels, which are low, moderate and high.

### 3.3 Ethical considerations

The research ethics committee at the University of Baghdad, College of Nursing consented the study proposal. All the collected data during the study course were recorded in such an approach that subject's identities remain confidential. A password‐protected file was used to store the study related‐electronic data. The collective form of the study findings is the only part that is disseminated in the study report. All the informed consents were signed by the subjects, where they had been informed that their participation is completely voluntary and they have the right to read, discuss and question the study protocol, the benefits and risks of participation with the researcher.

### 3.4 Statistical analysis

SPSS® version 20.0 was used to conduct the statistical analysis. Both descriptive and non‐parametric tests were employed to conduct the statistical analysis through the computation of the frequencies, percentages, Mann–Whitney test and Kruskal–Wallis test. The advantages of using non‐parametric tests can explain choosing them to be used in this study. Examples are however not limited to: their statistical potential to give meaningful results even when having a small sample size. Of equal importance, when compared with parametric tests, the non‐parametric tests constitute less strict assumptions, which make them more feasible option. Furthermore, their fixability to be used in many kinds of categorical, as well as interval data can make them a valid choice for fulfilling research endeavours. Finally, their ability to show the mean rank gives a very clear idea about the processed data in terms of their contribution in explaining the phenomenon under statistical investigation, as well as highlighting the significant statistical difference among the studied variables.

## 4 DISCUSSION

It is crucial to recognize that the transition from partial or complete dependence to full independence is a process that progresses gradually over time (Buscherhof, 1998). The quality and the speed of this progress depend on many factors. Examples are as follows: the condition's severity, availability of resources and most importantly the client's attitude in terms of adaption to neurologic deficits. Therefore, it is helpful to concede that complex situations which are related to stroke clients “vulnerability in terms of health‐illness transition, can be best explored through the lenses of nursing theories” (Meleis, 2010). For that reason, a directing theoretical framework is mandatory to understand stroke patient's response to their acute illness and guiding a tailored efficacious intervention based on their self‐efficacy level assessment. The transitions theory of Meleis's (2010) perfectly suits the study objectives. Meleis's definition of nursing is “the art and science of facilitating the transition of populations’ health and well‐being” (Meleis, 2010, p.5). According to this definition, nurse's professional identity can be actualized by holding the responsibility of being an advocator and facilitators of clients’ healthful transition towards ultimate psychosocial, spiritual, mental and physical well‐being. Gaining a deeper understanding of stroke patient's self‐efficacy in the acute phase of their health‐illness transition is essential to help them regaining their pre‐illness functionality. Based on this, it is mandatory to assess the patient's self‐efficacy level to design a tailored nursing intervention, which was addressed in this study through its first objective.

In her theory, Meleis emphasized that the dramatic stroke‐related pathophysiological changes can suddenly shift the client from wellness to critical sickness, which would not permit enough time for adaptation, especially during the acute stage of the illness. Therefore, she suggested that successful transition requires high level of resilience and self‐efficacy aiming at a meaningful redefinition of stroke clients’ identity in the transition time. Nursing therapeutics can be best approached when focusing on both the individual and the system levels (Lewin, Jöbges, & Werheid, 2013; Schumacher & Meleis, 1994). A tailored nursing therapeutic plan focusing on clients’ readiness, resources availability, enhancing adaptation capacities by working on resilience and self‐efficacy of the clients can be used to create an enabling environment that can promote healthy transition.

Meleis highlighted that personal meaning(s), expectations of the clients, level of knowledge or skill, environment, level of planning and emotional‐physical well‐being are major personal and environmental variables that can influence transitions (Meleis & Trangenstein, 1994). Therefore, the focus of this section would be discussing the study variables that might have contributed into stroke patients transition focusing mainly on socio‐demographic and clinical characteristics of patients with stroke and the significant statistical differences among them in light of the published literature.

In terms of sociodemographic characteristics, the finding of the study showed that more than half of patients were older adult males, which mirrors the stroke epidemiological models in the incidence literature. These findings come along with the results of Feigin, Lawes, Bennett, and Anderson (2003) where that they found that older adult males are more prone to stroke than females. However, Pierce et al. (2011) reported that while individuals’ vulnerability to stroke intensifies with increasing age, the medical literature reported that around 25% of strokes are affecting individuals who are younger than 65 years.

An alarming finding was emerged when highlighting the stroke‐related clinical information of patients recovering from stroke, which is that two thirds of the study sample were either completely unaware or uncertain about their specific stroke type, reflecting a knowledge deficit about their specific health problem and its physical and psychosocial consequences. On the other hand, this may also reflect healthcare provider's failure in addressing this knowledge deficit. All this may impair patient's compliance with preventive and rehabilitative endeavours (AL‐Fayyadh, & Mohammed, 2010). Becker et al. (2001) came into an agreement with current study results. This requires a mandatory change in care protocol aiming at getting patient on board as a real stakeholder in their care management. Of equal importance, the percentage of the study sample who were diagnosed with ischaemic stroke exceeded those with haemorrhagic type. This finding reflects the stroke global epidemiological models in the incidence literature, where ischaemic stroke is the highly prevalent type (Al‐Asadi & Habib, 2014; Benamer & Grosset, 2009).

**Figure 1:**
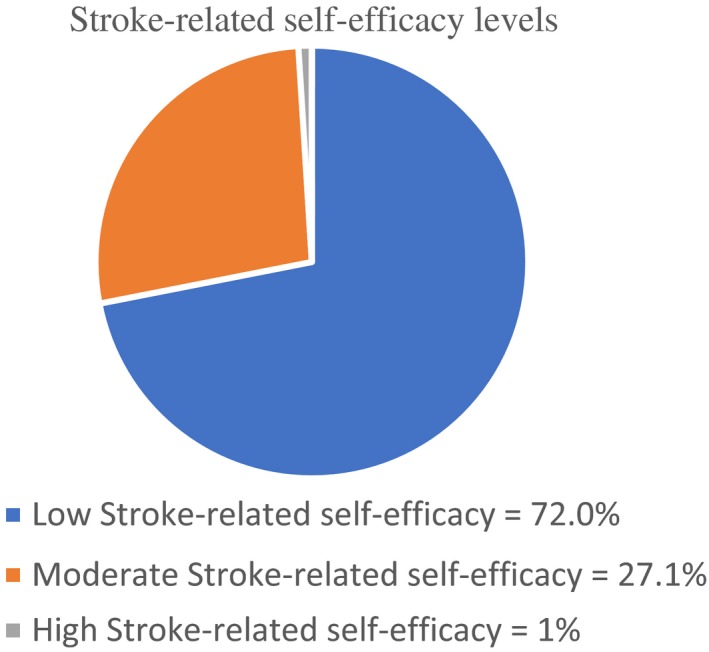
The highest percentage of the study subjects were classified under the category of lowest stroke‐related self‐efficacy, reflecting low functional independency. This was calculated based on the cut of point (0‐39) whereas 0 represents the lowest possible score, 39 is the highest possible score. Accordingly, Levels of self‐efficacy were classified in to the following: low = 0 ‐13, moderate = 13.1‐26, and high = 26.1‐39. The percentages of each level were represented by the pie chart to help readers seeing the whole picture of the study population in terms of their confidence level. Of equal importance, the mean score for level of subjects’ confidence of doing the tasks in spite of being affected by stroke was 10.48 signifying low overall self‐efficacy level

It is well documented in the relevant rehabilitation nursing literature that high‐level self‐efficacy of patients with stroke is connected with high‐quality ADL functioning (Korpershoek et al., 2011; Pang, Eng, & Miller, 2007; Robinson‐Smith, & Pizzi, 2003). Conversely, low self‐efficacy in stroke patients may decrease quality of life (Korpershoe et al., 2011). An unfortunate finding presented in Figure 1 shows the highest percentage of this research subjects were classified under the category of lowest stroke self‐efficacy, reflecting low functional independence. One way to explain this unfortunate finding is lack of holistic nursing care intervention, where nurses overlock the psycho‐social aspects of care plan. To address this defect, nurses need to value the self‐efficacy‐based intervention and its positive impact on improving stroke patient's functional capacity in terms of ADL and health‐related quality of life (Kvigne, Kirkevold, & Gjengedal, 2005).

Aiming basically at judging particular realms of poststroke independent functioning, such as Activities of Daily Living (ADL) and self‐management tasks. The study unidimensional tool was used to highlight the aforementioned realms. Reflecting on ADL, which are among the most basic and independent functions for human beings. However, they may pose a significant challenge to stroke patients. Payne, Stagnitti, Hooke, and Hitch (2015) reported that more than tree quartets of stroke patients may suffer from varying degrees of eating difficulties, which may jeopardize their independence of performing these basic activities. Despite this fact, persons are more likely to engage in a rigorous attempt to satisfy the luck of basic needs, such as hunger or thirst, aiming at achieving homoeostasis (Timmerman & Acton, 2001). Of equal importance, coping with stroke as an incapacitating disease can be highlighted by the multi‐faceted catastrophic effects of stroke on patient's life. In her theory's introductory framework, Meleis (2010) reported the feelings of desperation experienced by client, defining her stroke‐related hemiparesis as, “I feel like I have a dead body in bed with me” (p.27). Similarly, Sturm et al., (2004) captured stroke clients’ description about their life quality after being affected by this disease as, “equivalent to, or worse than, death” (p. 2342). All that may indicate transition failure in light of low stroke‐related self‐efficacy. Therefore, it is important to note that stroke clients are extremely vulnerable living under the dull shadows of their health problem, unless a specialized caring hand help them assuming more positive attitude facing the disease and smoothly transitioning towards fast and effective recovery. Nurses are integral to facilitate transitions, as they are key participants in the public health. Hartigan (2012) stressed that nurses represent the core of the stroke interdisciplinary rehabilitation team through their advanced communication skills that enable stroke clients to gain self‐awareness, self‐healing and “[empowering] the process of recovery through collaborative and meaningful integration of psychosocial needs, as well as physical functioning during the course of rehabilitation goal setting” (p. 69).

One principle objective of this study was to identify the differences in stroke self‐efficacy among some relevant variables. Highlighting theses statistical differences would be of great clinical value when designing stroke‐related self‐efficacy interventions. A significant statistical difference in stroke self‐efficacy was verified among subjects’ age, residency, stroke incidence and patient's knowledge about his/her stroke medical diagnosis. The literature showed that the dominant age group of clients with low self‐efficacy was less than 75 years (Andersson, Kamwendo, & Appelros, 2008). This comes along with the current study findings. Such findings can be best explained by the clinical fact that even minor strokes in senior person may lead to significant multisystem deficits, including cognitive, locomotive and sensory deficits. Which may influenced stroke patients goal attainment, outcome expectations and most importantly their self‐efficacy (Ireland & Arthur, 2006). This should not discourage healthcare providers to enhance older adult's self‐efficacy considering the fact that ageing is not a synonym of a disease. Instead, healthcare providers should deal with ageing as a biological process where they should support self‐efficacy aiming at achieving full independence (Rattan, 2014). Relative to the residency variable, Roos, Potgieter, and Temane (2013) have concluded that living environment has an impact on person's self‐efficacy level as the findings of their research revealed that metropolitans show up greater levels of self‐efficacy when equalled with countryside residents. This can be explained by that countryside residents had a perception of living in a poorer quality of life when compared with metropolitans. It is true that city living offers many advantages, such as better health care and its reflections on life quality; yet, rapid growing urbanization can intensify major health‐related risks and propose additional health hazards (WHO, 2010). Regarding to stroke incidence, the literature focused on presenting first time stroke patients experience, overlooking others who were affected repeatedly by it (Jones, Mandy, & Partridge, 2009; Omu & Reynolds, 2013). This finding justifies an in‐depth analysis of this segment of the targeted population. This low self‐efficacy in those with recurrent stroke can be explained by the poor life quality considering stroke tragic physical, social and psychological consequence (Hiraga, ; Wang et al., 2016). Finally, subjects who were knowledgeable about their medical diagnosis “stroke type” had better stroke‐related self‐efficacy than those who were completely unaware or uncertain about their specific stroke type. In their study, Denny, Vahidy, Vu, Sharrief, and Savitz (2017) concluded that self‐efficacy of stroke patients was positively affected by their knowledge about their health problem during the acute phase of their recovery. Marks, Allegrante, and Lorig (2005) explained that because self‐efficacy is modifiable in nature, thus knowledge may positively affect it in a way that can be reflected on stroke patient's clinical outcome. Therefore, they recommended that patient's literacy about their chronic conditions such as stroke must be enhanced in a way that boosts their self‐efficacy.

## 5 CONCLUSION

Low stroke self‐efficacy was evident predicting low level of autonomous functional capacities among the study sample. Subjects’ age, residency, knowledge about stroke specific medical diagnosis and stroke incidence were the most influential factors on the stroke patients related self‐efficacy, consequently on their functional independence during the recovery phase. Therefore, it is highly recommended that comprehensive rehabilitative programs should be tailored according to stroke patient's self‐efficacy, which reflects the accurate level of physical independence. Additionally, both self‐efficacy and its related functional independence are complex constructs that cannot explained by the contribution of single or mutable variables. In fact, they are the outcome of the interaction among the physical, psychosocial, demographic and clinical characteristics. Therefore, a mixed methods study is mandate considering its ability to highlight other variables that may contribute in explaining stroke self‐efficacy.

Being the patients official advocate, nurses can play a pivotal role with stroke patients by facilitating personal readjustment aiming at regaining full independence. However, under the current critical circumstances of Iraq, this would be challenging. Therefore, the WHO is invited to boost its valuable support to Iraqi nurses through mentoring and advocating nursing role in the multidisciplinary team of stroke patient care.

## CONFLICT OF INTEREST

None.
